# Correction: Meerz et al. Comparative Therapeutic Exploitability of Acute Adaptation Mechanisms to Photon and Proton Irradiation in 3D Head and Neck Squamous Cell Carcinoma Cell Cultures. *Cancers* 2021, *13*, 1190

**DOI:** 10.3390/cancers13236055

**Published:** 2021-12-01

**Authors:** Annina Meerz, Sara Sofia Deville, Johannes Müller, Nils Cordes

**Affiliations:** 1 OncoRay—National Center for Radiation Research in Oncology, Faculty of Medicine Carl Gustav Carus, Technische Universität Dresden, 01307 Dresden, Germany; annina.meerz@uniklinikum-dresden.de (A.M.); sarasofia.deville@onktherapeutics.com (S.S.D.); Johannes.Mueller@uniklinikum-dresden.de (J.M.); 2 Helmholtz-Zentrum Dresden-Rossendorf (HZDR), Institute of Radiooncology—OncoRay, 01328 Dresden, Germany; 3 German Cancer Consortium, Partner Site Dresden, German Cancer Research Center, 69120 Heidelberg, Germany; 4 Department of Radiotherapy and Radiation Oncology, University Hospital Carl Gustav Carus, Technische Universität Dresden, 01307 Dresden, Germany

The authors wish to make the following corrections to this paper [1]:

In the original article, there was a mistake in Figure 1F as published [1]. The presented data do not match the indicated scaling of the *y*-axis. In detail, the scatter plots of all cell lines, except for Cal33 cells, were plotted with a *y*-axis scaling from 0–40. This is incorrect, and the *y*-axis must be scaled from 0–80.

We have now corrected our manuscript accordingly and reformatted the affected cell lines in Figure 1F to a scaling from 0–80.

This solely formal mistake occurred due to a confusion of an earlier figure-version using different scaling while compiling and exporting the multipaneled figure. There is no need for any changes in the text as the correction does not affect any further parts of the study or any conclusions drawn from it. The corrected Figure 1 is shown below.

The authors apologize for any inconvenience caused and state that the scientific conclusions remain unaffected. The original article has been updated.

## Figures and Tables

**Figure 1:**
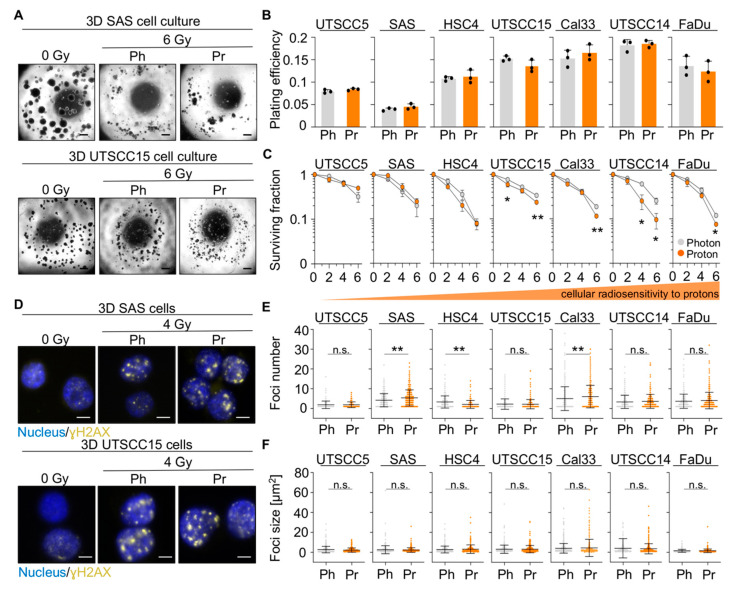
The intrinsic cellular radiosensitivity to photon and proton irradiation varies among 3D lrECM grown HNSCC cell cultures. (**A**) Representative microscopy images of unirradiated and irradiated 3D lrECM SAS and UTSCC15 cell colonies. Scale bar, 200 μm. (**B**) Plating efficiencies of unirradiated 3D lrECM HNSCC cell cultures. (**C**) Clonogenic radiation survival of indicated HNSCC cell lines upon photon or proton irradiation. (**D**) Representative immunofluorescence images of residual γH2AX foci at 24 h post 4-Gy irradiation (Scale bar, 20 μm; γH2AX in yellow and nuclei in blue). (**E**) Dot plots of residual foci numbers and (**F**) foci sizes 24 h post photon or proton exposure (4 Gy). At least 100 cells were analyzed per biological replicate. Results show mean ± SD (*n* = 3; two-sided *t*-test; * *p* < 0.05, ** *p* < 0.01). Ph, photon irradiation; Pr, proton irradiation; n.s., non-significant.
