# Supplementary material for: Comparative Therapeutic Exploitability of Acute Adaptation Mechanisms to Photon and Proton Irradiation in 3D Head and Neck Squamous Cell Carcinoma Cell Cultures
Source: Cancers (Basel). 2021 Mar 10;13(6):1190. doi: 10.3390/cancers13061190 (PMC8000891; doi:10.3390/cancers13061190)
Supplement: Supplementary file 1 [file cancers-13-01190-s001.pdf]

Comparative Therapeutic Exploitability of Acute Adaptation Mechanisms to Photon and Proton Irradiation in 3D Head and Neck Squamous Cell Carcinoma Cell Cultures

Annina Meerz[1], Sara Sofia Deville[1,2], Johannes Müller[1,2] and Nils Cordes[1,2,3,4*]

Supplementary Information

Supplementary Figures

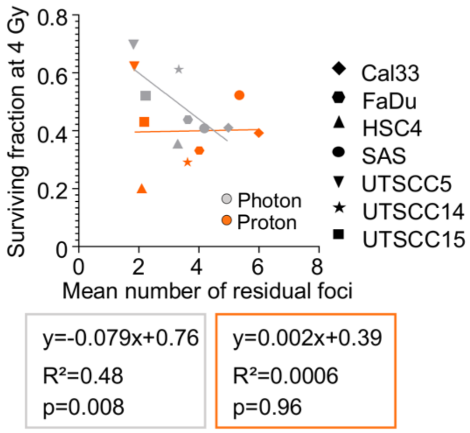

Figure S1. Correlation between mean foci number and surviving fraction at 4 Gy in examined 3D HNSCC cell cultures. Correlations between mean residual foci numbers and the mean surviving fractions at 4 Gy of either photon or proton irradiated 3D HNSCC cell cultures were calculated by linear regression.

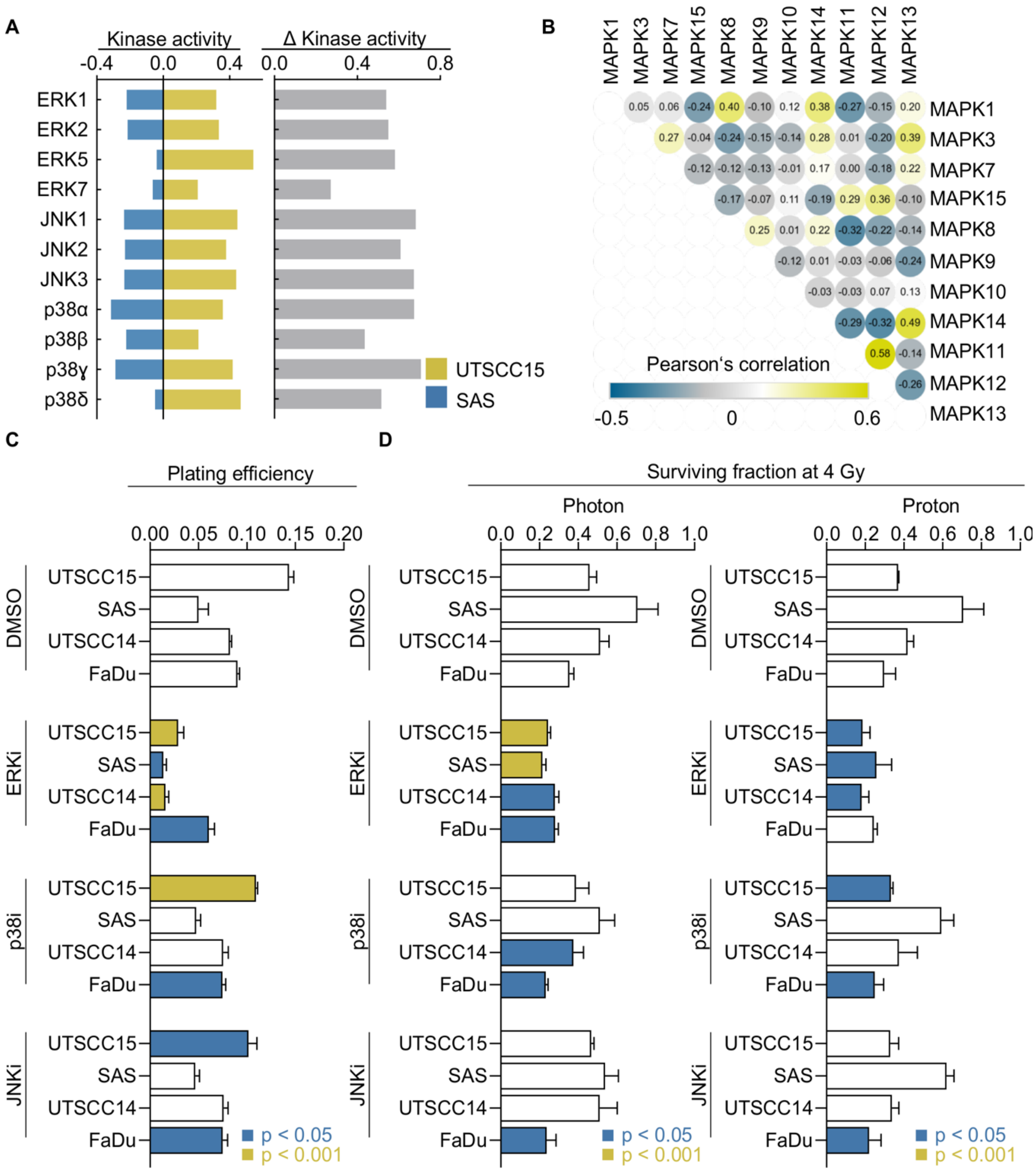

Figure S2. Differentially regulated mitogen-activated protein kinases upon proton and photon treatment and their role in radiosensitivity. (**A**) Comparative changes of indicated MAPK activities upon 4-Gy proton versus -photon treatment within the indicated cell lines 2 h post irradiation. (**B**) Correlation matrix of differentially regulated MAPK kinases mRNA expression. Each value represents the Pearson's correlation of the two genes. Positive correlations were shown in yellow and negative correlations in blue. Correlations were obtained from cBioportal. (**C**) Plating efficiencies and (**D**) clonogenic survival of indicated HNSCC cell cultures upon pretreatment with 3 different MAPK inhibitors (Ulixertinib for ERK1/2; SP600125 for JNK 1/2/3; Ralimetinib for p38α/β/γ/δ; all used at 1 μM). All results show mean ± SD (n=3, two-sided t-test).

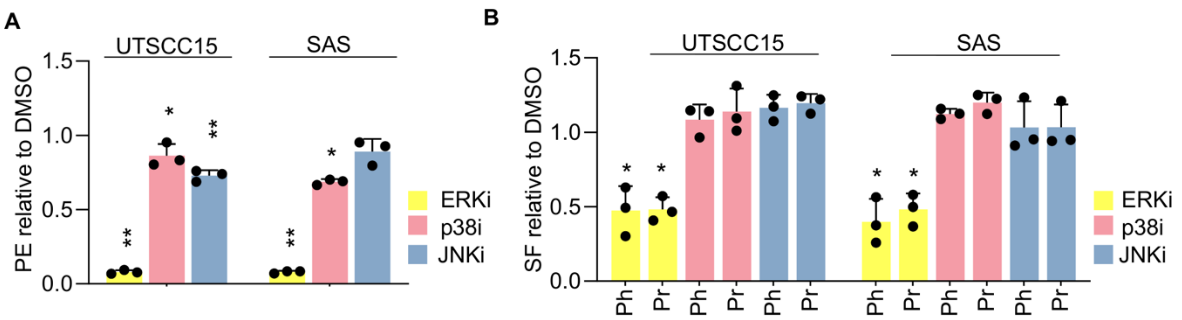

Figure S3. Effects of post-irradiation inhibition of MAPK signaling components in proton- or photon-irradiated 3D HNSCC cell cultures. (**A**) Normalized plating efficiencies and (**B**) clonogenic survival of UTSCC15 and SAS cell cultures upon inhibition of indicated MAPK (Ulixertinib for ERK1/2; SP600125 for JNK 1/2/3; Ralimetinib for p38α/β/γ/δ; all used at 1 μM). Inhibitor treatment was applied 30 min after 4-Gy irradiation exposure, respectively. DMSO served as control. All results show mean ± SD (n=3, two-sided t-test, *P<0.05, **P<0.01). Ph, photon irradiation; Pr, proton irradiation; PE, Plating efficiency; SF, Surviving fraction.

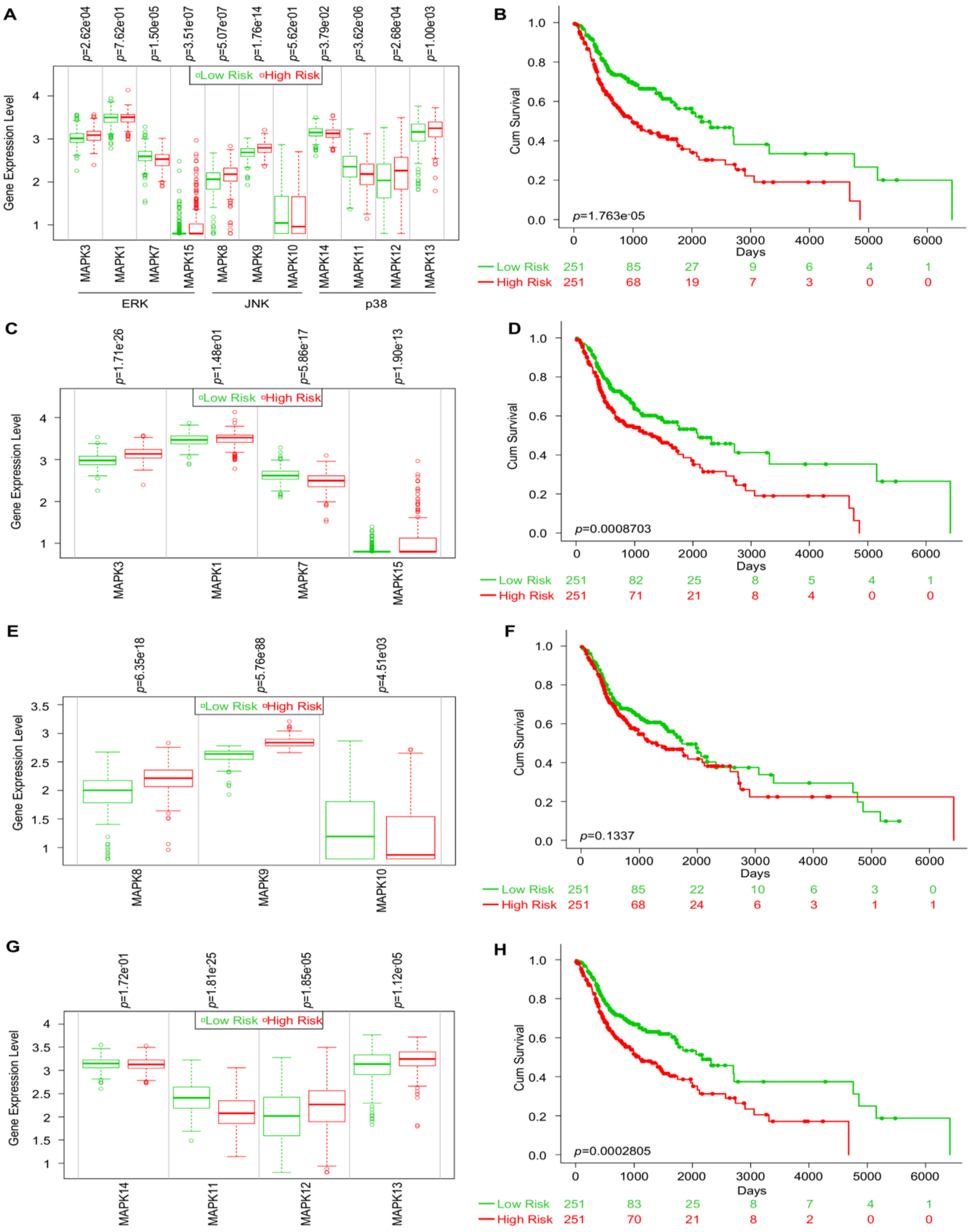

Figure S4. Expression levels of mitogen-activated protein kinases are relevant for patient survival. (**A**) Box and whisker plots of gene expression values of indicated MAPK kinases were compared between risk groups (low in green, high in red) by Student's t test. (**B**) Overall survival of risk groups (A) was evaluated by Kaplan–Meier survival plot and Log-Rank test. Total number of patients at risk were displayed at indicated time points (days). Box and whisker plots of (**C**) ERK kinases gene expression values (MAPK3 – ERK1, MAPK1 – ERK2, MAPK7 – ERK5, MAPK15 – ERK7), (**E**) JNK kinases gene expression values (MAPK8 – JNK1, MAPK9 – JNK2, MAPK10 – JNK3) and (**G**) p38 kinases gene expression values (MAPK14 – p38α, MAPK11 – p38β, MAPK12 – p38γ, MAPK13 – p38δ) were compared between risk groups by Student's t test. (**D, F and H**) Overall survival of risk groups (C, E and G) was evaluated by Kaplan–Meier survival plot and Log-Rank test. Total number of patients at risk were displayed at indicated time points.

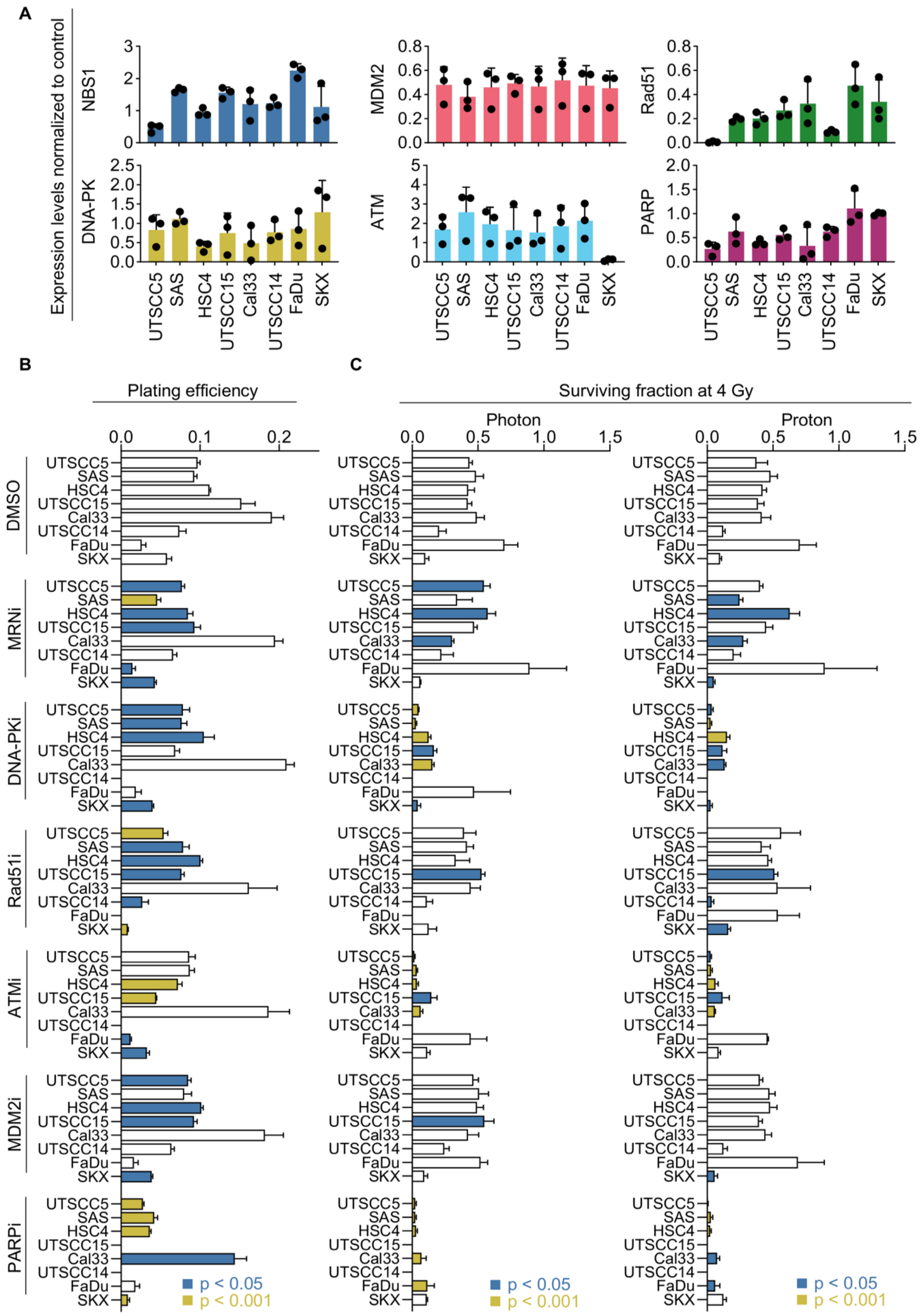

Figure S5. Uncovering the role of DNA repair proteins in the response to photon and proton irradiation. (**A**) Expression levels of indicated proteins upon normalization to β-actin. (**B**) Plating efficiency and (**C**) clonogenic survival of 3D HNSCC cell cultures upon 4-Gy photon or proton irradiation pretreated with indicated DNA repair inhibitors (Mirin for MRNi, NU7026 for DNA-PKi, B02 for Rad51i, KU55933 for ATMi, AMG232 for MDM2i, Olaparib for PARPi; all used at 10 μM). All results show mean ± SD (n=3, two-sided t-test).

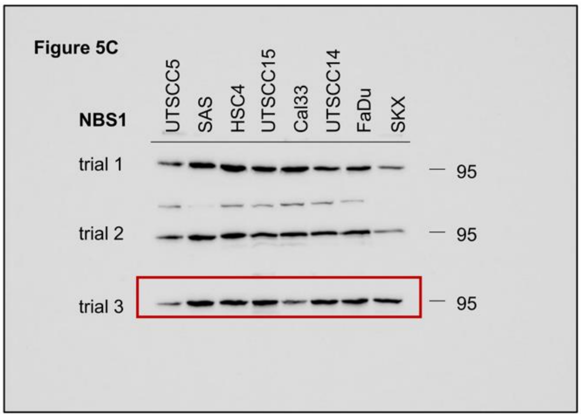
Figure 5C
NBS1
UTSCC5
SAS
HSC4
UTSCC15
Cal33
UTSCC14
FaDu
SKX
trial 1
95
trial 2
95
trial 3
95

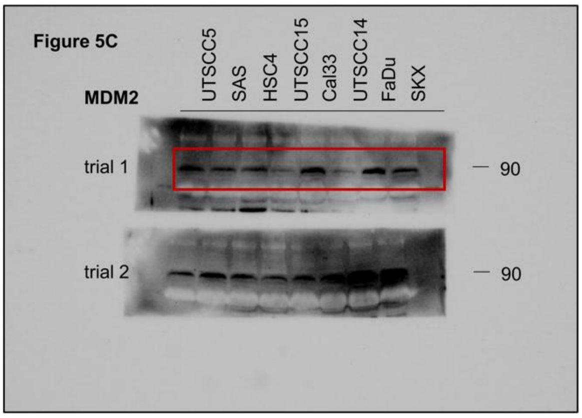
Figure 5C
MDM2
UTSCC5
SAS
HSC4
UTSCC15
Cal33
UTSCC14
FaDu
SKX
trial 1
90
trial 2
90

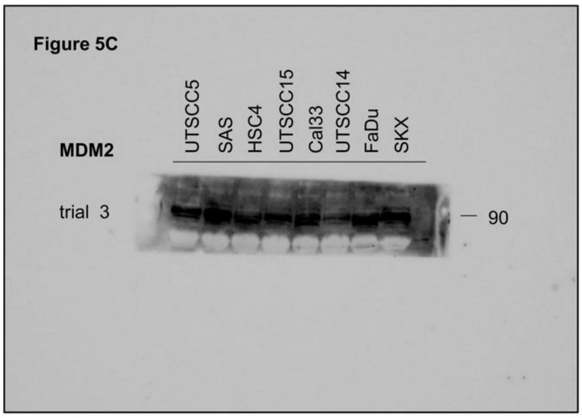
Figure 5C
MDM2
UTSCC5
SAS
HSC4
UTSCC15
Cal33
UTSCC14
FaDu
SKX
trial 3
90

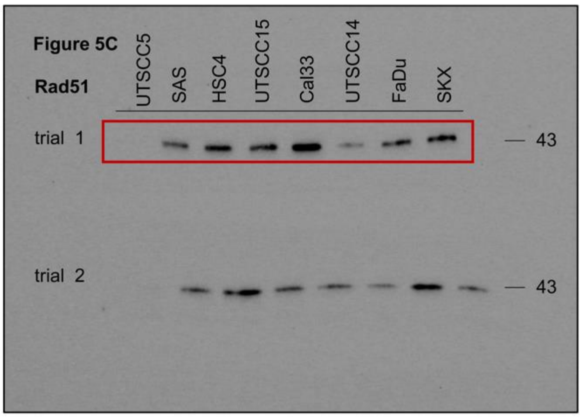
Figure 5C
Rad51
UTSCC5
SAS
HSC4
UTSCC15
Cal33
UTSCC14
FaDu
SKX
trial 1
43
trial 2
43

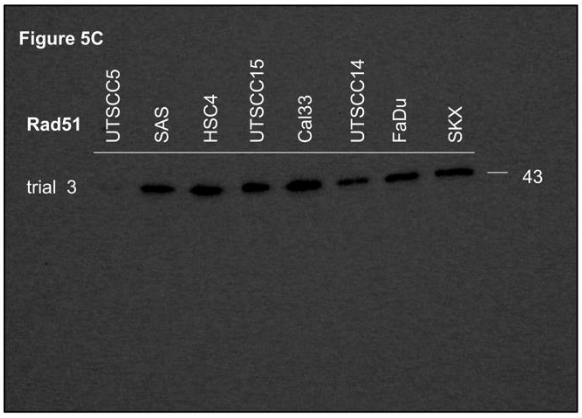
Figure 5C
Rad51
UTSCC5
SAS
HSC4
UTSCC15
Cal33
UTSCC14
FaDu
SKX
trial 3
43

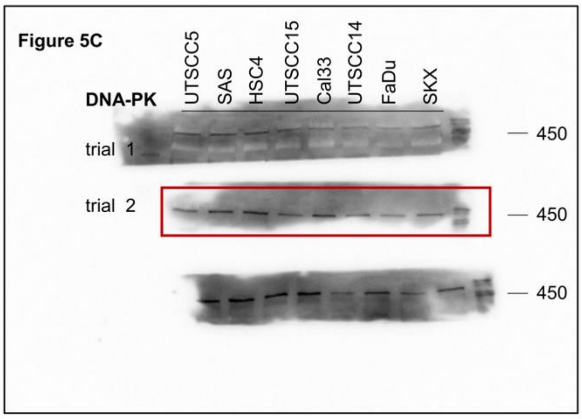
Figure 5C
DNA-PK
UTSCC5
SAS
HSC4
UTSCC15
Cal33
UTSCC14
FaDu
SKX
trial 1
450
trial 2
450
450

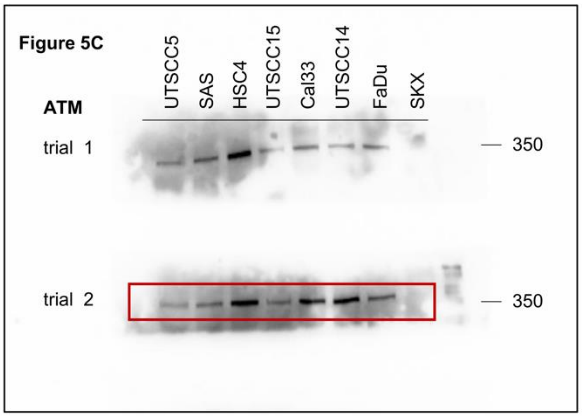
Figure 5C
ATM
UTSCC5
SAS
HSC4
UTSCC15
Cal33
UTSCC14
FaDu
SKX
trial 1
350
trial 2
350

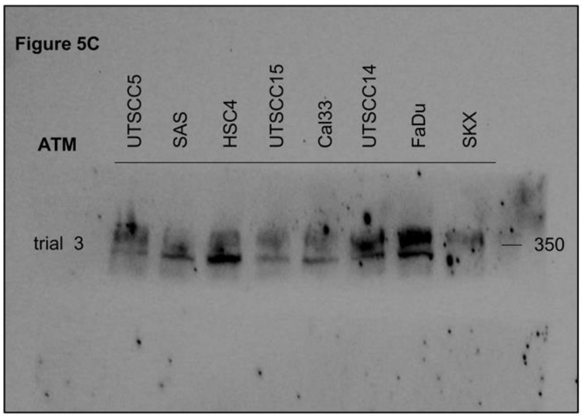
Figure 5C
ATM
UTSCC5
SAS
HSC4
UTSCC15
Cal33
UTSCC14
FaDu
SKX
trial 3
350

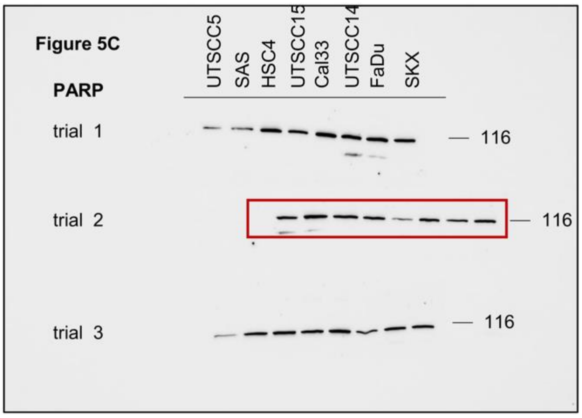

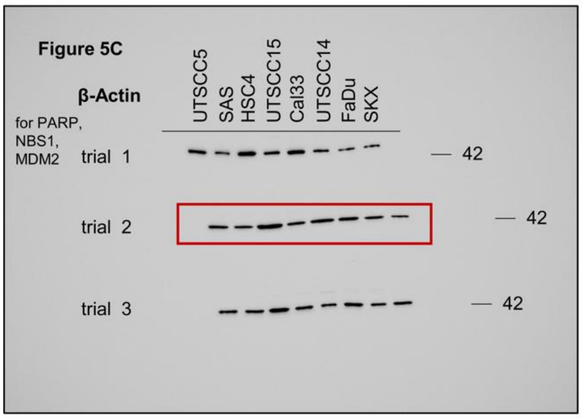

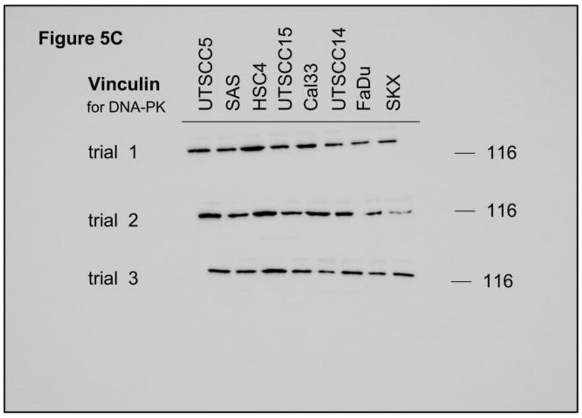

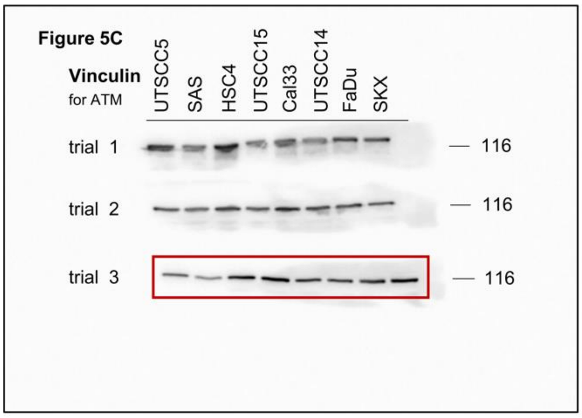

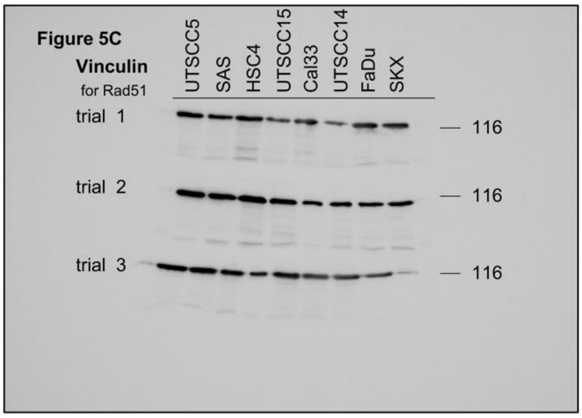

Figure S6. Compilation of uncropped Western blots for Figure 5C. The red rectangles display the areas of the plots that were used in Figure 5C. The molecular weight standard [kDa] per blot is located on the right. The detected protein is indicated on top of the blots.

Supplementary Tables

Table S1. Normalized densitometry readings of each band to control.

	NBS1		MDM2		Rad51	
	mean	SD	mean	SD	mean	SD
**UTSCC5**	0.456	0.129	0.481	0.148	0.006	0.007
**SAS**	1.641	0.071	0.382	0.114	0.195	0.021
**HSC4**	0.944	0.126	0.461	0.159	0.202	0.052
**UTSCC15**	1.556	0.173	0.492	0.076	0.269	0.077
**Cal33**	1.203	0.481	0.467	0.167	0.324	0.186
**UTSCC14**	1.227	0.160	0.517	0.185	0.092	0.014
**FaDu**	2.245	0.214	0.474	0.166	0.473	0.167
**SKX**	1.117	0.640	0.454	0.142	0.339	0.182

	DNA-PK		ATM		PARP	
	mean	SD	mean	SD	mean	SD
**UTSCC5**	0.831	0.389	1.693	0.714	0.270	0.140
**SAS**	1.113	0.164	2.577	1.296	0.629	0.278
**HSC4**	0.402	0.128	1.950	0.880	0.398	0.063
**UTSCC15**	0.750	0.517	1.643	1.183	0.557	0.125
**Cal33**	0.486	0.452	1.526	0.871	0.334	0.385
**UTSCC14**	0.772	0.286	1.845	1.075	0.629	0.106
**FaDu**	0.857	0.444	2.130	0.915	1.107	0.368
**SKX**	1.291	0.820	0.102	0.058	0.998	0.033
